# Chasing cortical behavior: designing multiphoton microscopes for imaging neuronal populations in freely moving rodents

**DOI:** 10.1117/1.NPh.10.4.044411

**Published:** 2023-10-25

**Authors:** Alexandr Klioutchnikov, Jason N. D. Kerr

**Affiliations:** Max Planck Institute for Neurobiology of Behavior, Department of Behavior and Brain Organization, Bonn, Germany

**Keywords:** fiber-optic applications, functional imaging, multiphoton microscopy, neurophotonics

## Abstract

Imaging in the freely moving animal gives unparalleled access to circuit activity as the animal interacts with its environment in a self-guided way. Over the past few years, new imaging technologies have enabled the interrogation of neuronal populations located at any depth of the cortex in freely moving mice while preserving the animal’s behavioral repertoire. This commentary gives an updated overview of the recent advances that have enabled the link between behavior and the underlying neuronal activity to be explored.

One of the advantages of recording neural activity in freely moving animals^1^ is that the animal has its full repertoire of sensory, proprioceptive, and vestibular feedback available^2^ and is free to interact with its environment as it determines. Although experiments with animals in various forms of restraint have the advantage of providing highly repeatable and stable experimental conditions, providing much of our detailed knowledge about cortical circuit function, restraining an animal severely limits the animal’s behavior and interaction with its surrounds. For example, the large eye rotations measured in freely moving rodents^3^ that counteract their head rotations enables their panoramic visual fields to remain stabilized in respect to gravity, even when pursuing prey,^4^ whereas head-fixed rodents have no appreciable eye motion. In addition, emerging neuronal functional properties, which were first discovered in the freely moving animal, such as cortical grid^5^ and hippocampal place cells,^6^ can be reproduced to some extent with a restrained animal using virtual reality setups,^7^ but significant differences in the neuronal responses have been found,^8^^,^^9^ emphasizing the utility of the freely moving animal experiments.

In the last decade, miniature one-photon excitation-based microscopes have been extensively used to image neuronal population activity using calcium indicators in freely moving mice.^10^ Such systems are relatively simple to use and come in many different designs, from wide field imaging of cortical areas^11^ to narrow field imaging, using GRIN lens, of deeper brain nuclei.^12^ Using single-photon excitation prevents optical sectioning, with single-cell resolution, in densely labeled tissue, and imaging depth from the surface is restricted (but see Ref. 13). As most head-mounted one-photon microscopes form images on a sensor, scattering of the emitted fluorescence degrades imaging quality, resolution, and signal-to-noise ratio (SNR)^14^ and does not allow for disambiguating neuronal identities enough for circuit reconstruction with single-neuron resolution.^15^ With the advent of nonlinear excitation-based microscopes such as two-^16^ and three-photon microscopes,^17^ combined with the spectacular array of genetically encoded fluorescent activity reporters,^18^ imaging neuronal populations has led to the link between the functional activity and cellular identification being explored.^19^ What makes this approach especially powerful is not only the ability to unambiguously identify the cell-type,^19^ spatial location,^20^ and generate multiple day activity recordings,^21^ but also, when combined with serial-block EM reconstruction, to model neuronal circuitry at the synaptic level.^15^^,^^22^ The goal of head-mounted miniature microscopes^10^^,^^23^^,^^24^ is to provide the ability to combine the advantages of imaging with the advantages of behavior in which the animal is free to interact with its environment in a self-determined way (for reviews see Refs. 25 and 26).

Miniature head-mounted multiphoton microscopes [Fig. 1(a)] are currently the only method to extract neuronal activity from cortical neuronal populations with enough spatial and temporal resolution to enable *post hoc* cell identity, with single-cell and subcellular resolution,^15^ in the freely moving animal (for review see Ref. 25). Since the first two-photon imaging of neuronal populations in the freely moving rodent,^24^ many technological advances have enabled not only further weight reduction that allows for imaging in the freely moving mouse^30^ and imaging the deep cortical layers^29^ or hippocampus^31^ but also minimal interference with the animal’s behavior through reducing the tethered burden.^32^

**Fig. 1:**
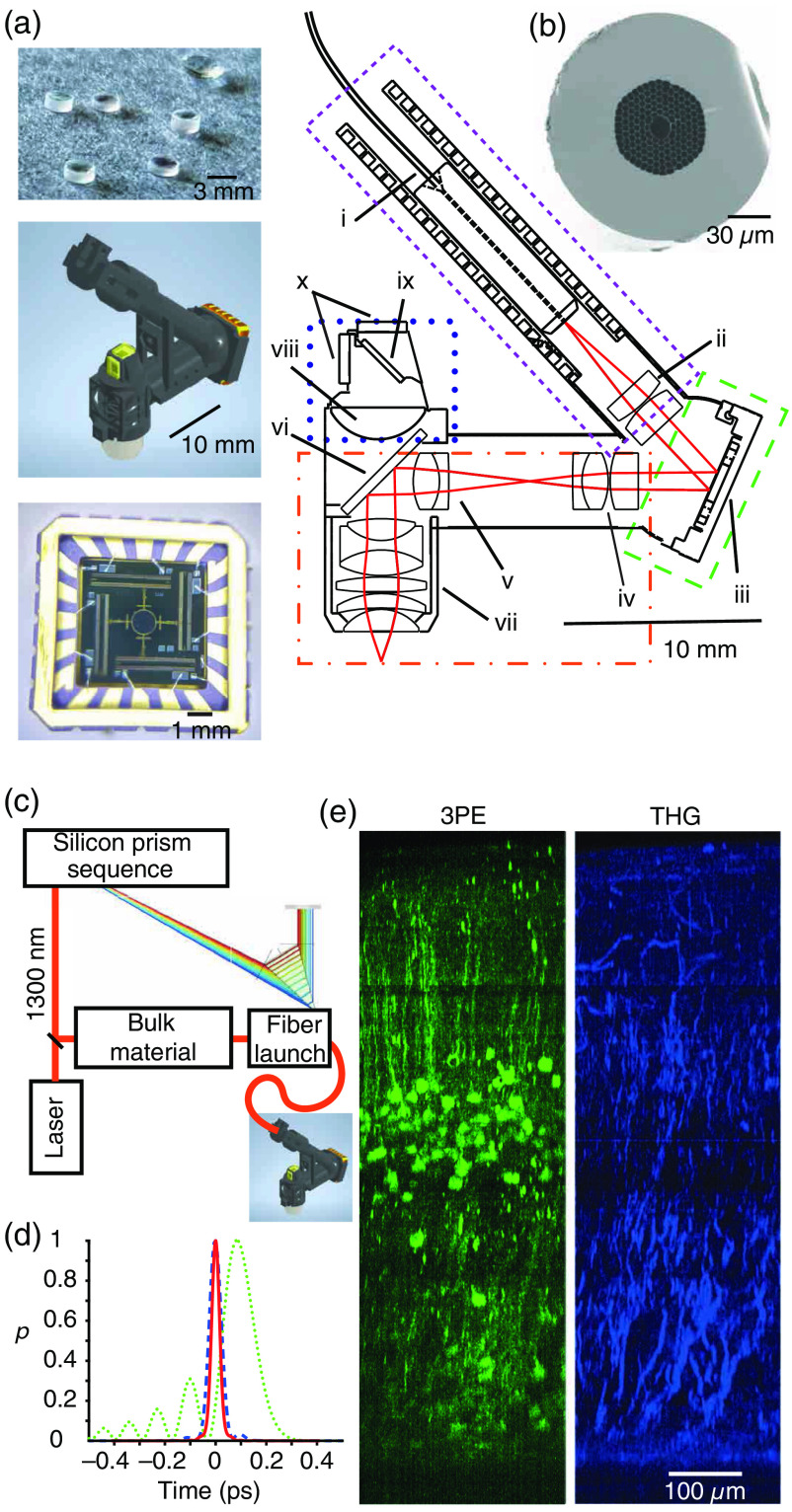
Miniature three photon microscopes enable imaging through the entire thickness of mouse visual cortex. (a) On the right, a cross-section of a typical miniature multiphoton microscope. Delimited are the laser delivery (small magenta dashes), laser scanning (green dashes), imaging optics (orange dash-dotted lines), and detection (blue dotted). The individual elements are from (i) to (x): (i) hollow core fiber with a ceramic ferrule glued at the end, (ii) collimation lens, (iii) MEMS scanner, (iv) scan lens, (v) tube lens, (vi) dichroic mirror, (vii) objective lens, (viii) collection lens, (ix) dichroic mirror, and (x)SiPMs. On the right from top to bottom are a photograph of miniature lenses, a CAD model of the whole microscope assembled, and a photograph of a MEMS scanner. (b) Electron micrograph cross-section of the hollow core fiber. (c) Schematic of the optical path and optical components for pulse distortion compensation. (d) Truncated laser pulse temporal profile after the fiber before dispersion compensation (green dotted) and after compensation (blue dashed) compared with calculated transform-limited pulse from the spectrum measured after the fiber (red continuous). (e) Side-projection showing jGCaMP7f-labeled neurons (left) and third harmonic generation signal (right) acquired using a mouse-capable three-photon miniature microscope from an anesthetized mouse. Scale in the right image applies to both images. The labeling was achieved by crossing two transgenetic expressing Cre recombinase in layer 4 (Ref. 27) and layer 6.^28^ Flexed GCaMP7f was introduced by an injection of a viral vector (AAV1/2.hSyn.FLEX.jGCAMP7f) to V1. Panels (c)–(e) are modified from Ref. 29 with permission.

The technical progress made with scanning approaches, such as the miniature electromechanical systems [MEMS, Fig. 1(a)],^33^ has allowed for access to large neuronal populations with frame rates fast enough to measure spiking activity patterns using genetically encoded calcium indicators (GECIs).^14^^,^^30^^,^^32^ Although two photon excitation (2PE) ensures that optical sectioning and conventional microscopes can form images to around 1 mm^34^ below the mouse brain surface,^35^ due to the smaller optics and scattering, the practical depth limit for miniature head-mounted 2P microscopes is around 400 to 500  μm.^14^ More recent microscope designs have taken advantage of three photon excitation (3PE) and have extended the imaging depth by reducing the out-of-focus fluorescence due to higher nonlinearity of excitation and lower scattering of deeper infra-red wavelengths.^29^

Efficiently eliciting 3PE of GECIs requires significantly higher instantaneous laser-power than for 2PE.^36^ Delivering the required laser intensity, typically tens to hundreds of nanojoules, has long been a challenge.^23^ The recently developed head-mounted 3PE microscopes have overcome this problem, first, using hollow core fibers [Fig. 1(b)] that cause significantly less distortion of the temporal profiles of light pulses due to their lower nonlinear effects compared with standard single-mode fibers.^37^ Second, second- and third-order dispersion has been compensated by introducing silicon prism sequences along with thick silicon slabs in the beam path. The final result is a temporal profile for the laser pulses of about 50 femtoseconds [Figs. 1(c) and 1(d)].^29^ This approach enabled imaging through the entire cortical column [Fig. 1(e)] in freely moving mice (Fig. 2).

**Fig. 2:**
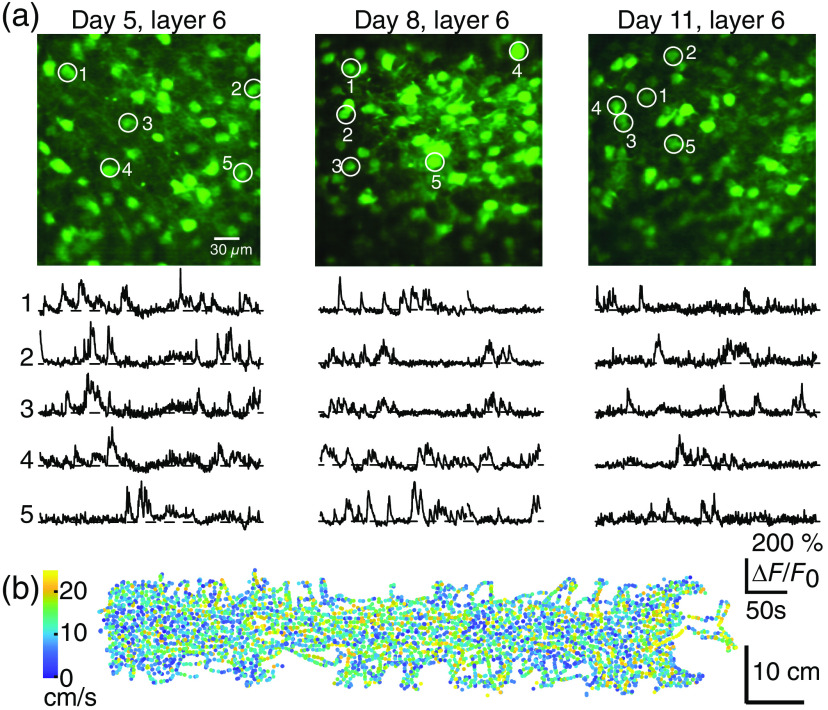
Miniature three photon microscopes enable imaging of cortical layer 6 in freely moving mice. (a) Imaging overview with 10 example neuronal calcium-fluorescence traces from different fields of view on imaging day 5 (left 5 days after implantation of the cranial window), imaging day 8 (middle) and imaging day 11 (right), for fields of view in cortical layer 6 of a freely moving mouse. All data from one animal, the same as imaged for the Z-stack in Fig. 1(e). Scale bar in left overview image applies to all overview images; scale bars between left and middle neuronal calcium traces apply to all calcium traces. Numbering of neuronal calcium traces corresponds to numbering of neurons in the corresponding overview image. The image is an average image of all frames acquired during the whole imaging session of about 5 min and motion corrected. (b) Example animal trajectory during one 20 min. Behavioral session for an animal carrying the miniature microscope, with position on the track color-coded by velocity. Panels (a) and (b) are modified from Ref. 32 with permission.

The underlying motivation for imaging neuronal activity in the freely moving animal is allowing the animal to behave in a self-determined manner, meaning that the design of the head-mounted devices needs to be tailored to minimally interfere with the animal’s behavior during data collection. This is an important constraint and requires not only that the total weight of the microscope is well below 10% of the animals’ weight but also that head-motion is not restricted. To achieve this a key challenge in the design of head-mounted microscopes is the management of cable-bundle flexibility.^14^ The plastic optical fibers (POFs) originally employed for conducting emitted light to the PMTs^24^ are too stiff to use for mice. Coherent fiber bundles, while providing a number of substantial advantages,^38^^,^^39^ are resistant to twisting. Although significant improvement in the flexibility of light collection fibers has been achieved using glass fiber bundles and tapering,^14^ a recent solution to minimize tether stiffness was to use on-board detection systems.^32^ These silicon photomultipliers (SiPMs) are light and small enough to be embedded into detector systems on the head-mounted microscopes and to significantly improve the SNR [Figs. 3(a)–3(c)]. In addition, as natural behavior includes various ambient light levels that are traditionally the enemy when imaging the brain with fluorescence-based microscopes, SiPMs readily support imaging under any light conditions. SiPMs are intrinsically resilient to ambient light exposure, unlike with the PMTs that are commonly used on conventional multiphoton microscopes that can be permanently damaged from ambient light exposure. SiPMs can be used in behavioral arenas requiring ambient light^32^ for behavioral pose tracking,^40^ eye tracking^3^ and visually based prey capture behaviors. In addition, their fast response currents also allow for fast temporal gating, clocked at the excitation laser pulse rate, for optical noise rejection [Fig. 3(d)] and to support temporal multiplexing for multiplane imaging.^41^

**Fig. 3:**
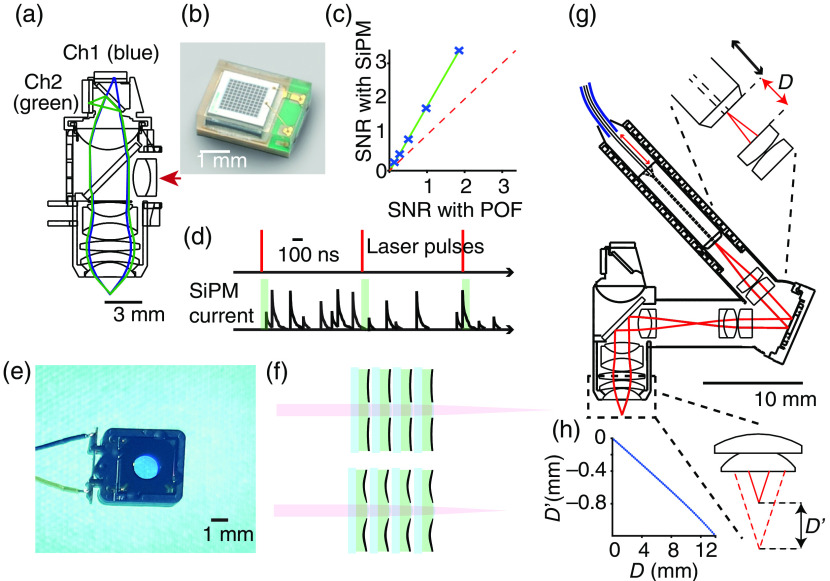
Recent technological advances enabling new capabilities in miniature multiphoton microscopes. (a) Microscope schematic highlighting the detector system, showing light paths and on-board detectors for green (green) and third harmonic generation signal (blue) channels. Scanner and z-drive optics omitted. Direction of excitation path indicated by red arrow. (b) Image of one of the on-board detectors (SiPM). (c) Image SNR measured using SiPM and remote PMTs coupled via a POF. SNR was measured with the same microscope and imaging the same fluorescent solution with only the detection featuring either SiPM or POF-PMTs. Signal defined as the average and noise as the standard deviation. Regions of interest of various sizes were used to generate multiple points (crosses). Unity plot shown in dashed red. (d) Schematic of timing of excitation pulses (upper) and emitted fluorescence integration window (lower, green boxes). (e) Photograph of a single microT Lens from poLight. (f) Principle of operation of a microTLens stack featuring four lenses stacked together as was used by Ref. 14 for remote focusing. Piezoelements bend a polymer lens as a function of applied voltage. This lens is typically inserted between the collimation lens and the MEMS scanner and ads little weight (<0.1  g) to the device. (g) Microscope cross-section showing a mechanical mechanism for remote focusing. Shifting the fiber-tip through distance D (top inset) shifts the image plane by distance $D^{\prime }$ (bottom inset). The fiber is slid through its jacket by a motor placed remotely. (h) Zemax-simulation of relationship between the lens-fiber tip distance (distance D in panel a) and corresponding image plane depth (distance $D^{\prime }$ in panel a). Panels (a)–(d), (g), and (h) are modified from Ref. 32 with permission.

Recent advances in remote focusing mechanisms for head-mounted microscopes have enabled changes in the imaging plane depth during free behavior,^14^^,^^32^^,^^39^ reducing the need to manually interact with the animal that the older focusing designs required.^24^ Two mechanisms for remote focusing have been implemented for moving the imaging plane in head-mounted microscopes, both using similar approaches that were originally established in larger table-mounted multiphoton microscopes.^42^^–^^44^ The first approach uses an electrotunable lens^14^^,^^39^ [Figs. 3(e) and 3(f)] that can change its optical properties, thereby changing the excitation focal point in the tissue, fast enough to image multiple planes.^14^ Given the current tunable range of this lens, this approach can change the focal plane up to 240  μm, which is enough to cover roughly a third of the cortical thickness. The second approach can change the focal plane of the image by taking advantage of the diverging excitation beam leaving the fiber tip [Figs. 3(g) and 3(h)]. This works by physically moving the fiber-tip with respect to the collimation lens and thereby controlling the amount of beam divergence or convergence (defocus) after the collimation lens. To achieve this, the fiber is pushed or pulled inside its jacket by a motor placed remotely from the microscope, on the optical table. This defocus is optically relayed to the MEMS scanner and then downstream to the objective lens, resulting in a focal range of >700  μm, while the excitation NA is maintained and optical aberrations are kept minimal. This is enough range to remotely focus through the entire mouse cortical column.^32^ Although providing a very substantial increase in the focusing range, this mechanical system has the drawback that the movement is much slower and is not suitable for fast multiplane imaging, but rather for sequential interrogation of populations in the same animal from any cortical layer.

Head-mounted microscopes allow for long-term recording from hundreds of cortical neurons over multiple days, and they can sample from neuronal populations spread throughout the cortical depth during a wide range of complex behaviors. Although specific distortion issues of miniature optics and scanners have been recently addressed,^14^^,^^45^ many parameters of miniature microscopes, such as scanning rates, optical resolution, imaging depth, and field of view, are unlikely to be improved significantly without major innovation (see Table 1). Future directions for this field could include leveraging off recent progress in large table-top multiphoton microscopes regarding multiplexing and simultaneous imaging multiple regions.^41^ Because recent advances in both head-mounted microscopes and genetically encoded indicators enable imaging from the same populations of neurons over months from freely moving mice, many of the more vexing neuroethological questions can be addressed, such as those requiring social interaction or learning paradigms spread over days. Over the next decade, head-mounted microscopes that provide optical sectioning will become an indispensable tool in the neuroethology toolbox by providing the opportunity to explore the link between behavior and neuronal activity in the freely exploring animal.

**Table 1:** Miniature microscope imaging parameters.

Work	Modality	Scanning	Detection	Optical resolution	Imaging depth	Speed	Field of view	Remote focusing
Zong et al.^14^	Two photon	MEMS	Remote PMT, flexible fiber	Lateral 1.2 μm, axial 13 to 18 μm	Not specified, cortical layer 2/3	40 Hz, 256 × 256 pixels	420 μm, square	Fast (0.4 ms), light (60 mg), 240 μm range
Klioutchnikov et al.^32^	Three photon	MEMS	On-board, SiPM, no fiber	Lateral 1.2 μm, axial 10 to 15 μm	All cortical layers, 800 μm	10.6 Hz, 273 × 280 pixels	300 μm, square	Slow, light (100 mg), 700 μm range
Zhao et al.^31^	Three photon	MEMS	Remote PMT, flexible fiber	Lateral 1 μm, axial 7 to 8 μm	CA1, 1200 μm	16 Hz, 128 × 128 pixels	400 μm, square	Fast (1.5 ms), 1.8 g, 180 μm range
Accanto et al.^38^	Two photon	Fiber bundle	Remote camera, stiff fiber bundle	Lateral 2 μm, axial 7 to 13 μm	150 μm	50 Hz	250 μm, circular	None

A comparison of the main imaging parameters of recently published miniature microscopes with single cell resolution and optical sectioning.

## Code, Data, and Materials Availability

No code, data, or materials have been used for this article.
